# *Staphylococcus xylosus* and *Staphylococcus aureus* as commensals and pathogens on murine skin

**DOI:** 10.1186/s42826-023-00169-0

**Published:** 2023-08-02

**Authors:** Michael Battaglia, Lee Ann Garrett-Sinha

**Affiliations:** Department of Biochemistry, Jacobs School of Medicine and Biomedical Sciences, State University of New York at Buffalo, Buffalo, NY 14203 USA

**Keywords:** *Staphylococcus aureus*, *Staphylococcus xylosus*, Skin, Infection, Virulence, Species-specific differences

## Abstract

Skin ulcers, skin dermatitis and skin infections are common phenomena in colonies of laboratory mice and are often found at increased prevalence in certain immunocompromised strains. While in many cases these skin conditions are mild, in other cases they can be severe and lead to animal morbidity. Furthermore, the presence of skin infections and ulcerations can complicate the interpretation of experimental protocols, including those examining immune cell activation. Bacterial species in the genus *Staphylococcus* are the most common pathogens recovered from skin lesions in mice. In particular, *Staphylococcus aureus* and *Staphylococcus xylosus* have both been implicated as pathogens on murine skin. *Staphylococcus aureus* is a well-known pathogen of human skin, but *S. xylosus* skin infections in humans have not been described, indicating that there is a species-specific difference in the ability of *S. xylosus* to serve as a skin pathogen. The aim of this review is to summarize studies that link *S. aureus* and *S. xylosus* to skin infections of mice and to describe factors involved in their adherence to tissue and their virulence. We discuss potential differences in mouse and human skin that might underlie the ability of *S. xylosus* to act as a pathogen on murine skin, but not human skin. Finally, we also describe mouse mutants that have shown increased susceptibility to skin infections with staphylococcal bacteria. These mutants point to pathways that are important in the control of commensal staphylococcal bacteria. The information here may be useful to researchers who are working with mouse strains that are prone to skin infections with staphylococcal bacteria.

## Background

In this review we seek to evaluate and curate the literature relevant to the staphylococcal species *Staphylococcus xylosus* and *Staphylococcus aureus* as commensals and pathogens on the skin of laboratory mice and to compare their properties to each other and to other common skin staphylococcal species such as *Staphylococcus epidermidis*. We also discuss potential virulence factors for these species and differences in skin structure that may contribute to the species-specificity of certain staphylococcal infections.

## Main text

### Staphylococcal species

Staphylococcus is a genus of bacteria containing roughly 60 species and subspecies that colonize a variety of environments [1]. Staphylococci are Gram-positive organisms in the phylum Firmicutes and they are characterized by formation of clusters of round cells (cocci). Staphylococcal bacteria are a significant component of the skin microbiome in many animals as observed in humans, mice, dogs, horses, cows, pigs, chickens and pigeons [2]. Some species of staphylococci are frequently associated with infections of the skin and underlying tissues, while other species are rarely associated with infection. This difference in pathogenicity between species is likely influenced by the presence or absence of a variety of virulence-associated genes in the different species. One of the traits used to divide staphylococcal species is based on expression of coagulase, an enzyme which can convert fibrinogen in serum into fibrin and hence coagulate the serum. Most strains of *Staphylococcus aureus* are coagulase positive, while other staphylococcal species commonly encountered on the skin, such as *S. xylosus* and *S. epidermidis*, are usually coagulase negative staphylococci (CoNS) [1]. Indeed, tests for coagulase activity are used clinically to help differentiate *S. aureus* from other staphylococcal species. In addition to coagulase positivity, numerous other characteristics have been used to differentiate staphylococcal species including the G + C content of the chromosome, the molecular composition of the cell wall, and patterns of antibiotic susceptibility, among other factors. This is complemented by 16S rRNA sequencing and whole genome sequencing that have allowed further refinement of the staphylococcal family tree. 16S sequencing led to the identification of 6 major staphylococcal species groupings, which can be further divided into fifteen clusters based largely on conservation and expression of factors such as coagulase, oxidase, and novobiocin resistance [3].

### Presence of staphylococcal species on human versus murine skin

In humans, the skin is colonized by several staphylococcal species, the most prevalent and widespread of which are *S. epidermidis* and *S. hominis* [2, 4, 5]. Other staphylococcal species can also be found on human skin, including *S. aureus*, *S. warneri*, *S. haemolyticus* and *S. capitis* [2, 5, 6]. On the other hand, *S. xylosus* is an infrequent colonizer of human skin, though it is more often isolated from the skin of people whose work brings them into frequent contact with animals [7]. When human skin is transplanted onto nude mice, *S. xylosus* can be recovered from the human skin grafts, but the percent of grafts colonized by *S. xylosus* is relatively low compared to the skin of host mice [8]. This implies that inherent features of murine skin promote the colonization by *S. xylosus*, while features of human skin discourage *S. xylosus* colonization. Many of the most common staphylococcal species on human skin fall into a sub-group of the staphylococcal species known as the Epidermidis–Aureus group [3]. On the other hand, *S. xylosus*, which is uncommon on human skin, falls into the Saprophyticus sub-group [3]. Among the human colonizing staphylococcal species, *S. aureus* has been most intensely studied, because it is one of the primary sources of skin and soft tissue infections (SSTI) [9–11]. Approximately 20–30% of humans carry *S. aureus* as a commensal on the skin or in the nasal cavity [5] and higher rates of nasal carriage of *S. aureus* are correlated with increased rates of *S. aureus* SSTI infections [12–14]. The other staphylococcal species that are present on the skin of humans are less frequently identified as the causative agents in infections. One study has shown that roughly 10% of SSTIs are caused by staphylococcal species other than *S. aureus* [15]. *Staphylococcus aureus* skin infections in humans can be serious and can invade into underlying tissues or enter the bloodstream, leading to life-threatening sepsis. These serious infections are complicated by the fact that some strains of *S. aureus* are resistant to antibiotics (e.g., the methicillin-resistant *S. aureus* (MRSA) strains).

A different set of staphylococcal species are common on murine skin with respect to human skin. On murine skin, the most prevalent staphylococcal species are *S. xylosus* and *S. sciuri* [2, 16, 17]. Microbiome studies of mouse ear skin from wild mice and C57BL/6 laboratory raised mice (both of the species *Mus musculus*) identified *S. xylosus* as a major colonizer [16], while human prevalent species such as *S. epidermidis*, *S. hominis* and *S. aureus* were not identified as a major species on murine skin in this study. Furthermore, *S. xylosus* has been repeatedly associated with infected skin wounds in mice (see Table 1), both in immunocompromised mice and in non-immunocompromised ones. *Staphylococcus xylosus* skin infections in mice lead to significant dermatitis often forming ulcers with serocellular crusts. When solutions containing *S. aureus*, *S. epidermidis*, *S. xylosus* or *S. lentus* were applied topically to the ear of C57BL/6 mice, *S. xylosus* was recovered at higher frequencies than the other Staph species when colony counts were obtained 2 days later [18]. These data suggest that *S. xylosus* preferentially survives or expands on murine skin. Furthermore, it is clear that *S. xylosus* is a frequent pathogen in murine skin, while it is very rarely the cause of pathogenic infections in human skin.

**Table 1:** Reports of *S. xylosus* skin infection in mice

Mouse strain studied	Potentially relevant defects	References
Forkhead box N1 (Foxn1) knockout (nude mice)	Lack of T cells and aberrant skin differentiation	[88, 89]
Neutrophil cytosolic factor 1 (Ncf1 or gp47^phox^) knockout	Deficient NADPH oxidase activity and impaired neutrophil function	[71–73]
Integrin beta 2 (Itgb2 or CD18) knockout	Defective neutrophil and T cell activity	[78]
Nitric oxide synthase 2 (Nos2) knockout	Deficient production of reactive nitrogen species, impaired neutrophil recruitment and delayed wound closure and increased Th1 responses	[76, 77]
Exogenous infection of SJL/J	No specific defect	[111]
C57BL/6 and a variety of knockouts on C57BL/6 background	No specific defect Defective production of IL-2, IL-4, IL05, IL-10, and/or IL-12 Defective complement Defective NK cell mediated antibody dependent cytotoxicity and phagocytosis Defective mast cell responses	[76]
Cytochrome b-245 beta chain (Cybb or gp91^phox^) knockout	Deficient NADPH oxidase activity and impaired neutrophil function	[71]
Stearoyl CoA desaturase 1 (SCD1) knockout	Aberrant skin differentiation	[36]
Replication timing regulatory factor 1 (Rif1) knockout	Impaired class-switch recombination of immunoglobulins	[92]
Nuclear factor of kappa light polypeptide gene enhancer in B cells inhibitor, zeta (Nfkbiz or IκBζ) knockout	Elevated serum IgE and increased numbers of IL-17A-secreting CD4+ T cells in skin	[69]
Recombination activating 1 (Rag1) and mitogen-activated protein kinase kinase kinase 8 (Map3k8 or Tpl2) double knockout	Lack of B cells and T cells and defects in superoxide production by macrophages	[87]
Leptin receptor (Lepr or db) knockout	Diabetic with chronic wound	[96]
Oxazolone induced atopic dermatitis lesions in C57BL/6J	Mixed Th1-Th2-mediated allergic response	[67]

The situation with *S. aureus* on murine skin is complicated. *Staphylococcus aureus* can be recovered from murine skin, but not all mouse colonies seem to carry *S. aureus* [19, 20]. Because not all mice carry *S. aureus*, it has sometimes been regarded as an organism transferred from human caretakers, rather than a commensal of mice. Complicating this, staphylococcal species including *S. aureus* and *S. xylosus* can spread rapidly among cohoused animals from animals [21] and hence transfer of *S. aureus* from caretakers to one or a few animals could lead to spread throughout the colony. However, in addition to potential transfer from caretakers, recent evidence shows that some colonies of mice carry mouse-adapted strains of *S. aureus* that differ from the strains common on human skin and these mouse-adapted strains are passed from murine parents to progeny, indicating a commensal relationship [19]. Similar to the observances in humans, colonization by mouse-adapted *S. aureus* in murine models appears to show a bias towards colonization of the nares [20, 22]. *Staphylococcus aureus* is a causative agent of skin infections in mice [23, 24], though reports of *S. aureus* skin infections in mice appear to be rarer than reports of *S. xylosus* skin infections. However, this may reflect a publication bias and further studies are needed to understand the true prevalence of *S. aureus* versus *S. xylosus* skin infections in mice. The observations described above show that the staphylococcal bacteria on mouse and human skin have different patterns of species prevalence. The differences in staphyloccocal colonization between mice and humans are likely due to inherent properties of both the bacterial species and the host skin environment.

### Properties of skin staphylococcal species

As described above, *S. xylosus* and *S. aureus* belong to different sub-groups of the Staphylococcus family tree [3]. However, they display many similarities in the mechanisms of skin colonization and their potential to become pathogenic. In terms of virulence factors, little is known about *S. xylosus*, since it is not a common human pathogen. However, genomic analysis of various *S. xylosus* isolates has identified a number of loci with significant homology to known virulence factors identified in *S. aureus*. Table 2 lists virulence factors described in *S. aureus* and indicates whether similar proteins have been identified in three other species of staphylococcal bacteria that colonize the skin of mice or humans, *S. xylosus*, *S. epidermidis* and *S. sciuri*. *Staphylococcus xylosus* possesses genes homologous to all of the *S. aureus* virulence factors listed in Table 2, although it should be noted that most of these genes have not been directly shown to have a pathogenic role in *S. xylosus*. On the other hand, *S. epidermidis* and *S. sciuri* have only been reported to contain a sub-set of these genes. This may imply that *S. xylosus* has more pathogenic potential than *S. epidermidis* or *S. sciuri*. These data should be interpreted with caution though, since future studies may identify additional virulence genes in one or more of these species. In addition, it is important to keep in mind that different bacterial isolates show differences in terms of the presence or absence of virulence factors, in part driven by the fact that some virulence factors are encoded on mobile genetic elements and can be transferred horizontally. Hence not all isolates of the species listed in Table 2 may contain all the virulence factors described. Despite these limitations, the current data support the idea that *S. xylosus*’ mechanisms of virulence may be similar to those described in *S. aureus*.

**Table 2:** Presence of virulence factors in Staph species

Virulence factor (*S. aureus*)	*S. epidermidis*	*S. xylosus*	*S. sciuri*
Autolysin (atl/atE)	[112]	[26, 113]	Not reported as yet
Biofilm associated protein (bap)	[114]	[26]	[115]
Elastin binding protein (ebpS)	Not reported as yet	[26]	Not reported as yet
Fibronectin binding protein (fnb)	Not reported as yet	[26]	[115]
Laminin binding protein (eno)	[116]	[26]	[116]
Methicillin resistance protein (fmt)	Not reported as yet	[26]	Not reported as yet
mecA or mec homolog (mecC)	[117]	[113, 118, 119]	[120]
Toxic shock syndrome toxin (tst)	Not reported as yet	[118, 121]	[122]
Staphylococcus enterotoxins	[123]	[38, 124, 125]	[115]
Hemolysin (hla, hlb, hld, hlg)	[123]	[126]	[127]
Panton-Valentine leukocidin (lukS-PV, lukF-PV)	Not reported as yet	[128]	Not reported as yet
Exfoliative toxins (eta, etb, etd)	Not reported as yet	[38, 129]	[130]
Superoxide dismutase (sod)	[131]	[132]	Not reported as yet
*S. aureus* surface protein (sasC, sasG, sasX)	[133]	[27]	Not reported as yet
Phenol soluble modulins (PSMα, PSMβ, PSMδ, PSMε, PSM-mec)	[134–136]	[39, 137]	[137]

Some of the virulence genes shown in Table 2 include those that promote the adherence and invasion of tissue by *S. aureus*. These include microbial surface components recognizing adhesive matrix molecules, or MSCRAMMs for short, that bind to host extracellular matrix factors such as elastin, fibronectin, and laminin. Both *S. xylosus* and *S. aureus* can generate biofilms, which aids in their persistence following initial colonization. Production of these biofilms is largely dependent on biofilm associated protein (bap) and sas/sxs proteins [25–28]. Interestingly, *S. xylosus* pre-colonization of murine skin reduces the ability of *S. aureus* to colonize and treatments that deplete commensal staphylococcal species, including *S. xylosus*, increase the colonization ability of *S. aureus* [29]. These data indicate that *S. xylosus* and *S. aureus* may compete for binding to similar ligands on the skin’s surface. Coculture experiments investigating biofilm formation of *S. xylosus* and *S. aureus* found that formation of *S. aureus* biofilms was inhibited in the presence of cell-free supernatants derived from *S. xylosus*, resulting in formation of *S. aureus* aggregates that were more susceptible to detachment [30]. These observances suggest that *S. aureus* and *S. xylosus* may occupy similar niches in vivo. However, it is likely that the differences in the structure and function of mouse and human skin as well as differences in bacterial physiology contribute to the differential presence of these bacteria on skin in humans and mice.

Despite being most often described as a extracellular pathogen, *S. aureus* can internalize into epithelial cells by a mechanism in which bacterial fibronectin binding proteins (FnBPs) bind to host fibronectin, which subsequently interacts with host α5β1 integrin followed by endocytosis of the complex [31]. *Staphylococcus aureus* cells can survive for some time inside mammalian cells, with survival being noted for up to 96 h in a human skin keratinocyte cell line [32] and up to 7 days in a human umbilical endothelial cell line [33]. The prolonged survival of *S. aureus* within mammalian cells may result in a reservoir of bacteria that is hidden from certain immune responses and that can escape the activity of antibiotics [31, 34, 35]. *Staphylococcus xylosus* can also internalize into cells as shown by a study with NIH3T3 fibroblasts [36]. *Staphylococcus xylosus* has a homolog of the FnBP proteins, but it is not yet clear if *S. xylosus* internalization takes place via a FnBP-fibronectin-α5β1 integrin pathway or not. These data suggest that both *S. aureus* and *S. xylosus* may have the ability to form persistent infection by hiding within cells in the skin environment.

Some *S. xylosus* isolates express staphylococcal enterotoxins, toxic shock syndrome toxin 1 and exfoliative toxins, which are virulence factors found in many strains of *S. aureus* [37, 38]. Similar to the corresponding virulence factor in *S. aureus*, *S. xylosus* phenol soluble modulins (PSMs) have been shown to be highly functional [39]. PSMα from *S. aureus* has significant cytolytic activity against erythrocytes, mast cells, and neutrophils isolated from both murine and human hosts. Similarly, PSMα proteins from *S. xylosus* are highly pro-inflammatory with greater observed neutrophil calcium flux than *S. aureus* derived δ toxin and PSMα3. In addition, *S. xylosus* PSMα is able to induce similar mast cell degranulation as *S. aureus* PSMα3. Despite the pronounced in vitro results, epicutaneous application of PSM-expressing *S. xylosus* only resulted in minor pathogenicity in a mouse atopic dermatitis model [39]. In SKH-1E female mice, an atopic dermatitis like condition develops upon application of δ-toxin expressing *S. aureus*. Infecting these mice with *S. xylosus* did not lead to similar atopic dermatitis like lesions and mutants of the PSM genes in [39] had no effect on the phenotype observed [39]. This latter result indicate that mice may be adapted to colonization by *S. xylosus* and do not induce a strong inflammatory response to the bacteria, despite production of functional PSM proteins.

Iron is a rate-limiting nutrient in infection settings and *S. aureus* scavenges iron from the host via a number of mechanisms, including the production of hemolysins, which are proteins that lyse erythrocytes and release iron-containing hemoglobin [40]. *Staphylococcus xylosus* expresses a functional delta-hemolysin protein [41] and hemolytic activity has observed in nearly 90% of *S. xylosus* isolates tested [42]. *Staphylococcus aureus* also uses siderophores, such as staphyloferrin A and B [43, 44], to take up free iron from the environment via the *fhu* system, which mediates the uptake of the siderophores [45]. Genomic analysis of the C2a strain *S. xylosus* indicates the presence of genes encoding staphyloferrin A as well the FHU system [46]. Therefore, *S. xylosus* may scavenge iron from the environment using mechanisms similar to those described in *S. aureus*. Cumulatively, current data suggest that *S. xylosus* and *S. aureus* share expression of homologous proteins, which are known to contribute to virulence in *S. aureus*, though the role of most of these virulence factors in natural *S. xylosus* infections in mice remains to be tested.

### Skin structure in mice and humans and relevance to staphylococcal colonization/infection

Skin serves as a major interface between the body interior and the exterior environment, where many microbes are found colonizing the skin’s surface. The basic cellular structure and organization of the skin of humans and mice has many similarities, but there are also key differences that distinguish the two species and that are relevant to studies using mouse models. In both mice and humans, the skin is composed of an epidermal layer, a dermal layer and a hypodermal layer. The epidermal layer is primarily composed of stratified squamous epithelial cells termed keratinocytes that undergo a differentiation program to produce a protective barrier that excludes bacteria and other contaminants, while also preventing the excess loss of fluids from underlying tissues. The dermis of both species is largely composed of fibroblasts and the extracellular matrix they secrete. The dermal compartment also contains blood vessels, immune cells, nerve endings, sweat glands, sebaceous glands and the bulbs of hair follicles. The hypodermis is composed primarily of fat cells and connective tissue and serves a cushioning function.

Despite these similarities in overall structure, there are a number of fundamental differences between the skin of mice and humans. One significant difference is in the thickness of the skin. In humans, the epidermis is composed of 5–10 layers of keratinocytes, whereas mice only have 2–3 layers of keratinocytes [47]. In addition, mice and humans differ significantly in the number of hair follicles [48], with mice having a significantly higher density and a more even distribution of hair follicles than humans. The hair shafts in mice are also thicker than human body hair, which is very fine [49]. On the other hand, humans have a significantly greater number of eccrine sweat glands that are distributed over the entire surface, while mice have sweat glands only on their paws. Human skin is attached to the underlying tissue, while murine skin is loose. Human skin contains rete ridges, projections of the epidermis that extend into the underlying dermis, while murine skin lacks rete ridges. Another key difference is the presence of the panniculus carnosus, a sub-cutaneous muscle layer underlying the entire skin in mice, but which is largely absent in human skin with the exception of a vestigial presence in specific regions [50].

Resident immune cells are found in the skin in both the epidermal and dermal compartments. Innate lymphocytes (ILCs), neutrophils, macrophages, mast cells, natural killer cells, and epidermal Langerhans cells are found in the skin of both mice and humans [51, 52]. In addition, both mice and humans have αβ and γδ T cells in the skin [53]. In human skin, T cells are primarily located in the dermis and mostly carry the αβ T cell receptor. Human skin also has a small proportion of skin resident γδ T cells [47]. Like human skin, murine skin has dermal αβ and γδ T cells. However, one significant difference in the immune makeup of murine versus human skin is the presence of a specialized type of γδ T cells in murine skin, called dendritic epidermal T cells (DETC) [48]. As their name suggests, they are found in the epidermal layer of the skin and have a dendritic morphology. DETC express an invariant T cell receptor composed of Vγ5 and Vδ1 chains and are involved in skin inflammatory and wound healing processes [54]. Their functions in humans are likely carried out by multiple other cell types such as Vδ1+ and Vδ2+ γδ T cells, which share various functions including modulating wound healing and secretion of effectors such as IFNγ and IGF-1 [55, 56].

Some of the differences in structure between mouse and human skin may contribute to colonization by different staphylococcal species, although this has not been directly tested. An example of a possible structural difference that may be relevant is the differences in sweat gland distribution in mice and humans. As noted above, human skin has many more eccrine sweat glands distributed over the entire body, while murine skin has eccrine sweat glands only on the paws. The enzyme lysozyme, which breaks down bacterial cell walls, is produced by human sweat glands [57]. *Staphylococcus aureus* is known to be resistant to the effects of lysozyme [58], while *S. xylosus* is not [59]. Thus, the presence in humans of large numbers of sweat glands secreting lysozyme might lead to conditions that are unfavorable for *S. xylosus* colonization, while permitting *S. aureus* colonization. Another potentially relevant factor is that human skin has fewer hair follicles than murine skin. Studies in mice lacking EGF receptor in keratinocytes have shown overgrowth of *S. xylosus* and the presence of gram-positive bacteria in the hair follicles [60]. If *S. xylosus* preferentially colonizes hair follicles, it may be better able to colonize animal skin where there are more hair follicles than human skin. While neither of these potential mechanisms has been experimentally validated, it is possible that these and other differences in mouse and human skin underlie the differential ability *S. xylosus* to colonize this tissue.

### Mouse susceptibility to skin staphylococcal infections

**Fig. 1:**
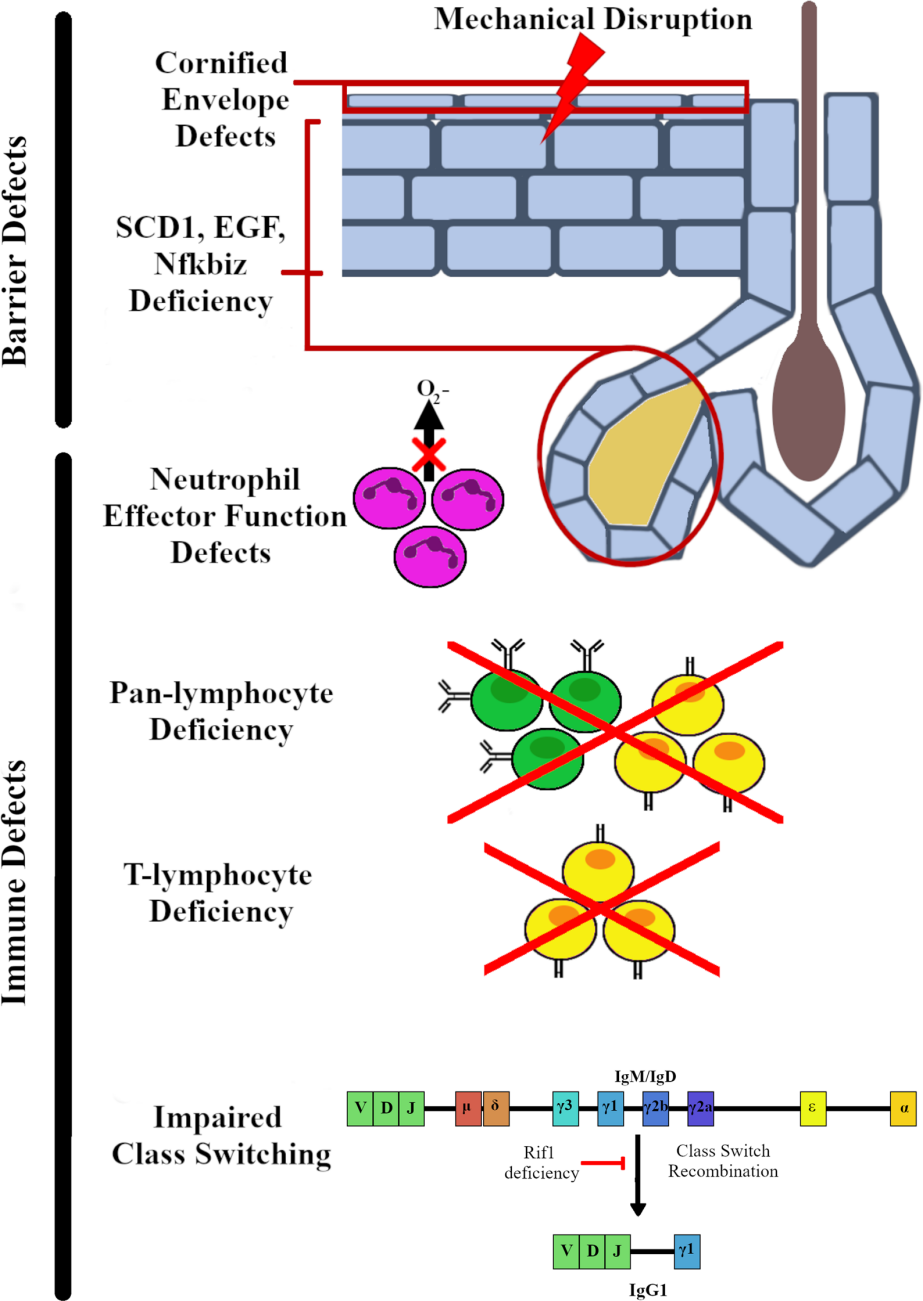
Mechanisms of susceptibility to *S. xylosus* spontaneous skin infection. Spontaneous infections of murine models of disease reveal specific mechanisms important for prevention of *S. xylosus* establishing infection in skin. These can be broadly broken down into two classes, barrier and immune related. Mice with barrier function defects including those with defects in the cornified envelope and altered structure of the stratified keratinocyte layers show a significant susceptibility to spontaneous *S. xylosus* infection. In addition, mice with impaired reactive oxygen species production, lacking lymphocytes, lacking T lymphocytes, or displaying impaired class switching indicate immune mechanisms important for the normal immune response to *S. xylosus*

*Staphylococcus xylosus* is an uncommon infectious agent in humans and for this reason it has not been studied as a pathogen using mouse models. Therefore, data concerning the immune response to *S. xylosus* is derived from spontaneous infections that occur in particular mouse strains. Published studies identifying *S. xylosus* as the causative agent in spontaneous murine skin infections are outlined in Table 1. These reports show that mouse models where there is a disruption of the skin barrier are often susceptible to *S. xylosus* infection. In addition, specific defects in innate and adaptive immune cell responses can also lead to susceptibility to skin infection with *S. xylosus*. On the other hand, spontaneous *S. aureus* skin infections in mice are more rarely reported and much of the data concerning pathways that regulate mouse susceptibility to *S. aureus* come from studies where the bacteria have been exogenously applied, epicutaneously (on the surface of the skin), intradermally (injected) or into open wounds on the skin. Infection of the deeper layers of the skin, such as the intradermal model of infection, typically leads to formation of an abscess, while infection of the superficial layers of the skin does not. Several recent reviews have described the immune response to *S. aureus* infection of murine skin, including important cell types and effector molecules [61–63]. For this reason, we focus here on the immune mechanisms that appear to be important for responses to *S. xylosus*. These pathways are also summarized in Fig. 1.

### Skin barrier function defects

The skin forms a barrier between the external environment, where bacteria are found, and the internal tissues. Defects in this skin barrier confer susceptibility to staphylococcal infections. Mechanical disruption of the skin barrier in mice using tape stripping to remove the superficial layers that form cornified envelopes results in increased colonization and persistence by epicutaneously applied *S. aureus*, with a more significant effect seen in animals with increased levels of tape stripping compared to animals with mild tape stripping [64]. Similarly, in mice lacking the cornified envelope protein filaggrin, tape-stripping and ovalbumin (OVA) sensitization resulted in significant barrier function impairment and *S. aureus* invasion of the dermis and underlying adipose tissue following epicutaneous application [65]. Studies indicate that an intact skin barrier is also crucial for prevention of spontaneous *S. xylosus* skin infections [21]. Treatment of murine skin with the chemical oxazolone results in an epidermal barrier defect caused by degradation of filaggrin and E-cadherin [66]. These oxazolone-treated mice show striking increases in *S. xylosus* skin colonization [67]. Spontaneous skin infections with *S. xylosus* have also been described in mice lacking the stearoyl-CoA desaturase (SCD1) enzyme, which is required for generation of lipids involved in establishing a water-permeability barrier in the skin [36]. The susceptibility to staphylococcal infection in SCD1 deficient mice also extends to *S. aureus*, since mice with a recessive germ line mutation in SCD1 display significantly impaired clearance of intradermally injected *S. aureus* [68]. The epidermal growth factor (EGF) is important for establishing the skin barrier and mice with a tamoxifen-inducible keratinocyte-specific deletion of the EGF receptor develop a barrier defect that initiates in the hair follicles [60]. These mice also demonstrate an overgrowth of *S. xylosus* on the skin. Mice lacking the NFκB cofactor Nfkbiz (IκBζ) also develop spontaneous skin infections with *S. xylosus* [69]. Nfkbiz can be induced in many cell types including both immune cells and epithelial cells in response to TLR signaling. In the skin, Nfkbiz is also expressed at the basal state in keratinocytes surrounding the hair follicle [70]. Nfkbiz deficient mice have both a skin barrier defect and an overgrowth of *S. xylosus* on the skin [69]. Together, these studies show that a variety of mechanical and genetic manipulations that affect the skin barrier lead to increased colonization and persistence of staphylococcal bacteria.

### Defects in neutrophil functions

Bacterial infection leads first to activation and recruitment of innate immune cells, such as monocytes, macrophages and neutrophils. Neutrophils are particularly important in the control of bacterial infections, since they are the most abundant white blood cell type and they phagocytose bacteria and kill them. Neutrophils are involved in generating abscesses in response to bacterial infection and this process is dependent on the NADPH oxidase complex that generates toxic reactive oxygen species that can kill bacteria. NADPH oxidase activity is crucial for control of staphylococcal infections as shown by the fact that mice lacking components of the NADPH oxidase complex, p47phox (Ncf1) or p91phox (Cybb or Nox2), are susceptible to spontaneous skin infections by *S. xylosus* [71–73]. NADPH oxidase is also required in humans to prevent staphylococcal skin infection, since patients with chronic granulomatous disease (CGD) that have mutations in the NADPH oxidase NOX2 subunit show enhanced susceptibility to *S. aureus* skin infections [74]. Production of reactive nitrogen species in neutrophils by the action of the enzyme inducible nitric oxide synthase 2 (Nos2) is also involved in bacterial killing [75]. Mice lacking Nos2 also show a susceptibility to spontaneous skin infections with *S. xylosus* [76, 77]. Finally, mice lacking the integrin subunit Itgb2 (also called CD18), which is expressed on a variety of different white blood cells including neutrophils and macrophages, are susceptible to spontaneous skin infections by *S. xylosus* [78]. All these studies point to the crucial role for neutrophils and the innate immune response in the control of *S. xylosus* skin infections.

### Adaptive immunity

T cells are crucial for control of skin *S. aureus* infections in mice and γδ T cells that produce IL-17 have been particularly implicated in this process using mouse models. However, different mouse models have yielded somewhat disparate and sometimes contradictory results based on the strain of mice and the infection model used. For many studies of *S. aureus* pathogenesis, mice of the C57BL/6 genetic background have been used. In studies using C57BL/6 mice, loss of γδ T cells, but not αβ T cells, led to impaired clearance of primary intradermal *S. aureus* infections [79]. In the skin, γδ T cells are the primary source of the cytokine IL-17 [80], and IL-17 and IL-17 receptor are both also required for optimal clearance of murine skin infections by *S. aureus* [79–81]. Loss of IL-17 receptor signalling in humans also leads to susceptibility to skin Staphylococcal infections [82]. Supporting an important role for γδ T cells in anti-Staphylococcal responses, RNA-sequencing of draining lymph nodes at 28 days post-infection showed expansion of a particular TCR Vγ6 and TCR Vδ4 sequences, while there was not strong enrichment for specific αβ TCR sequences [80]. Some studies in C57BL/6 wild-type mice show a minimal protection afforded by a primary *S. aureus* skin infection and secondary infection results in similar lesion sizes and bacterial burdens [83, 84]. However, in another study, C57BL/6 mice did show protection in a secondary infection, though this was due to an innate immune response since a similar protective effect was observed in Rag1−/− mice [85]. The reasons for the discrepancy in secondary responses of C57BL/6 mice in these studies are not apparent, but may be due to differences in experimental protocols such as the dose, timing and site of infection. C57BL/6 mice with a deletion of IL-1β were shown to have a worse primary response with increased bacterial burden, but a normal secondary response suggesting that there was development of immunological memory [83]. The protective response in IL-1β-deficient mice was shown to be mediated by γδ T cells producing TNF and IFNγ and to not require specific antibodies [83].

C57BL/6 mice are known to have a bias towards developing Th1 cells [86], leading to increased IFNγ secretion. On the other hand, Balb/c strain mice have a Th2 bias and develop more T cells secreting IL-4 [86]. When studying Balb/c mice with *S. aureus* skin infections, differences were found in comparison to studies using C57BL/6 mice. In Balb/c mice, the protective responses to secondary infection were found to be significantly stronger than in C57BL/6 mice [84, 85]. This memory response in Balb/c mice was dependent on CD4 T cells, which drive production of protective antibody responses [84, 85]. On the other hand, C57BL/6 mice don’t develop protective antibody under the same conditions. IL-17 is required for the secondary responses to repeat infections by *S. aureus* in Balb/c mice, while IFNγ in C57BL/6 mice blocks a protective effect [84]. The differing results obtained in different conditions indicate that careful attention should be paid to experimental variables such as mouse strain and infection protocols in order to best determine the roles of adaptive immunity in Staphylococcal infection.

Few studies have exogenously infected mice with *S. xylosus* and hence most of the data concerning immune responses relevant to *S. xylosus* clearance are derived from spontaneous infections. Rag1-/- mice, which completely lack B cells and T cells, have been shown to be susceptible to spontaneous *S. xylosus* skin infection [87]. Similarly, nude mice, which lack T cells due to a thymic defect caused by mutation of Foxn1, are also susceptible to spontaneous skin infections by *S. xylosus* [88, 89]. However, the latter observation should be interpreted cautiously because the Foxn1 protein also regulates keratinocyte growth and differentiation in the skin and hair follicles [90]. Hence, nude mice may show susceptibility to *S. xylosus* either because they lack T cells or because they have aberrant skin differentiation or both. Topical infection of skin with *S. xylosus* in wild-type mice elicits significant recruitment CD8+ T cells, whose recruitment accelerates wound healing [91]. B cells may also have a role in controlling *S. xylosus* infections. Mice lacking the Rif1 gene, which is involved in B cell class-switch recombination, develop skin infections with *S. xylosus* [92]. This may indicate that specific Ig classes are more effective in controlling *S. xylosus* infection and that class switching is required for bacterial clearance.

### Diabetes

Human patients with diabetes are susceptible to development of diabetic foot ulcers on the skin of the feet [93] and these are often colonized by *S. aureus* [94]. *Staphylococcus aureus* infection of diabetic foot ulcers is associated with delayed wound healing. Patients with diabetes also tend to show higher rates of nasal carriage of *S. aureus* [95]. Together these observations suggest that diabetes may either promote the growth and attachment of staphylococcal bacteria and/or impair the immune response to the bacteria.

Interestingly, studies of the wound microbiome in diabetes prone db/db mice, which lack the leptin receptor, found that *S. xylosus* was often one of the first and most prevalent colonizers in a skin wounding model and that its colonization was strongly associated with development of a chronic wound [96]. Similarly, wild-type C57BL/6 mice where diabetes was induced by injection of streptozotocin had deficiencies in clearing skin infections with *S. aureus*, which could be reversed by treating with prostaglandin E2 that induces dendritic cell dependent induction of Th17 cells [97, 98]. Streptozotocin-induced diabetes also impairs healing of *S. aureus* infected skin wounds in rats [99].

A model of human diabetic foot ulcers has been developed which involves the injection of *S. aureus* bacteria into the hind footpad of mice, with or without diabetes. db/db mice injected in the hindpaw with *S. aureus* show defects in bacterial killing accompanied by reduced neutrophil respiratory burst leading to chronic infections [100]. Similarly, non-obese diabetic (NOD) mice with diabetes and wild-type C57BL/6 mice where diabetes was induced with a high fat diet also show impaired clearance of *S. aureus* in the hindpaw injection model [101, 102]. The susceptibility observed in high fat diet induced diabetic mice was attributed to significantly reduced expression of *Aicda,* which induces somatic hypermutation and class switch recombination in B cells, likely contributing to significantly decreased IgG and IgE responses in diabetic mice versus controls [101]. Cumulatively, these findings indicate that diabetes contributes to susceptibility to staphylococcal infections, including infections of the skin.

## Conclusions

In this review, we’ve summarized reports of the bacterial species *Staphylococcus xylosus* as a cause of skin infections in mice, including laboratory mice used in research. *Staphylococcus xylosus* is a commensal of murine skin, but can become pathogenic when there are disruptions to the skin barrier and/or when the host immune response is compromised. The presence of diabetes is also a risk factor for *S. xylosus* infections. *Staphylococcus aureus* can also serve as both a commensal and pathogen of murine skin, but not all strains of mice carry *S. aureus* and there are fewer published reports describing skin infections with *S. aureus* than with *S. xylosus*. Unlike in mice, *S. xylosus* rarely causes infections of the skin or other tissues in humans, while *S. aureus* is a well-known and important pathogen in humans.

*Staphylococcus xylosus* strains express many genes with significant similarity to known virulence factors of *S. aureus*, but for the most part, the roles of these genes in *S. xylosus* infection have not been studied. In addition to its roles in mice, *S. xylosus* is also a common pathogen of food animals, including cattle [103], goats [104] and trout [105]. *Staphylococcus xylosus* can also be recovered from pet animals, such as dogs and cats [2, 106–109] and at least one instance of human infection with *S. xylosus* caused by dog bite has been reported [110]. Given the potential of *S. xylosus* to cause infections in research animals, food animals and pets and the possibility that such infections may be transmitted to humans, it would be of benefit to better understand the pathological mechanisms employed by *S. xylosus* that allow it to colonize tissues and persist in the presence of immune responses.

## Data Availability

No experimental datasets were collected for this study.

## Abbreviations

CoNS: Coagulase-negative staphylococci
SSTI: Skin and soft-tissue infection
MRSA: Methicillin-resistant *Staphylococcus aureus*
MSCRAMMS: Microbial surface components recognizing adhesive matrix molecules
FnBP: Fibronectin-binding protein
PSM: Phenol-soluble modulin
ILC: Innate lymphoid cell
DETC: Dendritic epidermal T cell
OVA: Ovalbumin
SCD1: Stearoyl-CoA desaturase
EGF: Epidermal growth factor
NADPH: Nicotinamide adenine dinucleotide phosphate
CGD: Chronic granulomatous disease
